# PPH Butterfly: a novel device to treat postpartum haemorrhage through uterine compression

**DOI:** 10.1136/bmjinnov-2016-000144

**Published:** 2017-01-02

**Authors:** Caroline Cunningham, Peter Watt, Nasreen Aflaifel, Simon Collins, Dot Lambert, John Porter, Tina Lavender, Tony Fisher, Andrew Weeks

**Affiliations:** 1 Women's and Children's Health, University of Liverpool, Liverpool, UK; 2 Medical Physics and Clinical Engineering, Royal Liverpool and Broadgreen University Hospitals NHS Trust, Liverpool, UK; 3 Interplex Select Moulds, High Wycombe, UK; 4 EPS Ltd, Wirral, UK; 5 University of Manchester School of Nursing Midwifery and Social Work, Manchester, UK; 6 Royal Liverpool and Broadgreen University Hospitals NHS Trust, Liverpool, UK

**Keywords:** Postpartum Haemorrhage, Bimanual Compression, PPH Butterfly, Uterine Atony, Treatment

## Abstract

**Objective:**

Postpartum haemorrhage (PPH) is a significant cause of maternal morbidity and mortality. The most common cause is an inability of the uterus to contract adequately after childbirth. In bimanual compression (BMC), one hand is placed within the vagina and the other hand is on the abdominal wall to compress the uterus. It is effective, but very uncomfortable for the woman. We designed a device that could replicate BMC without inserting a hand vaginally, therefore being less invasive. It could also help in diagnosing the source of the bleeding.

**Design:**

Mixed methods, combining an iterative design process with input from clinicians in simulations, and focus groups of clinicians and consumers.

**Setting:**

Department of Women's and Children's Health and Department of Medical Physics and Clinical Engineering, University of Liverpool, UK.

**Methods:**

A multidisciplinary team developed the design, using an obstetric manikin. Clinician and consumer groups also gave input on the concept and design. A healthcare product company and prototype manufacturer provided input into strategy, design and manufacture.

**Results:**

The PPH Butterfly is a single piece, plastic medical device that replicates BMC. It is designed to be easy to use and low-cost and allows for smooth insertion and removal. It is acceptable to clinicians and consumers and performs well in tests.

**Conclusions:**

This is the first device designed to replicate BMC while being less invasive. It could potentially be an effective form of PPH management, while also diagnosing the source of the bleeding. The device will now be tested in humans.

## Introduction

Postpartum haemorrhage (PPH) is an obstetric emergency that can follow vaginal or caesarean delivery. It is estimated that each year PPH accounts for 27% of the 303 000 maternal deaths worldwide^1^ ^2^ and a further 20 million women suffer long-term effects.^3^ Although the absolute risk of death is much lower in high-income countries (1 in 100 000 vs 1 in 1000 births in low-income countries),^4^ and in spite of marked improvements in management, PPH remains a significant contributor to maternal morbidity and mortality throughout the world.^5^

A low-cost, effective intervention that can be used by first-level maternity services providers could be a major advance in reducing maternal mortality from PPH, especially in low resource settings where the majority of deaths occur.

A vital step in the physiological prevention of PPH is the immediate contraction and retraction of myometrial muscle fibres during and after the third stage of labour. Uterine atony is a condition characterised by the inability of the uterus to contract adequately after the placenta has separated from the uterus. This condition is thought to be the most common cause of PPH^6^ ^7^ and is often unpredictable.^8^ Its presentation is very similar to that of genital trauma and often the only way to distinguish the two is under anaesthetic in an operating theatre. Initial treatment therefore assumes that the cause of any postpartum bleeding is atony, with formal examination under anaesthetic only if this treatment fails. However, there is evidence that repeated use of uterotonics has little effect.^9–11^ Furthermore, on-going haemorrhage caused by delays in stopping the bleeding can lead to coagulation problems, which only serves to worsen the bleeding and sets up a vicious cycle. Given all this, it is fortunate that most women with PPH stop bleeding spontaneously.^12^ However, the inability to predict who will stop bleeding spontaneously forces caregivers to intervene aggressively at an early stage in the process on all women.

## The theory behind the device

Bimanual compression (BMC) is an old technique in which the uterus is compressed between a hand on the lower abdomen and a hand inserted into the vagina and formed into a fist. Although it is highly effective, it is a very painful manoeuvre for the mother (unless she has an epidural in place) and tiring for the practitioner. The idea of the Butterfly device is to achieve the benefits of BMC without being so invasive, thus allowing it to be more widely used. It has been designed to be a slim, easily insertable replacement to a fist in the vagina, thus increasing acceptability of uterine compression to women and clinicians.

**Figure 1:**
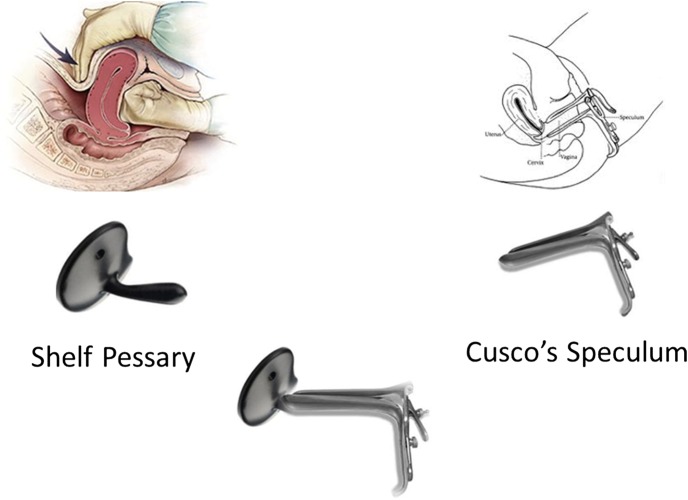
A composite picture combining a shelf pessary (used for the treatment of uterovaginal prolapse) and a Cusco speculum (used for vaginal and cervical examination).

**Figure 2:**
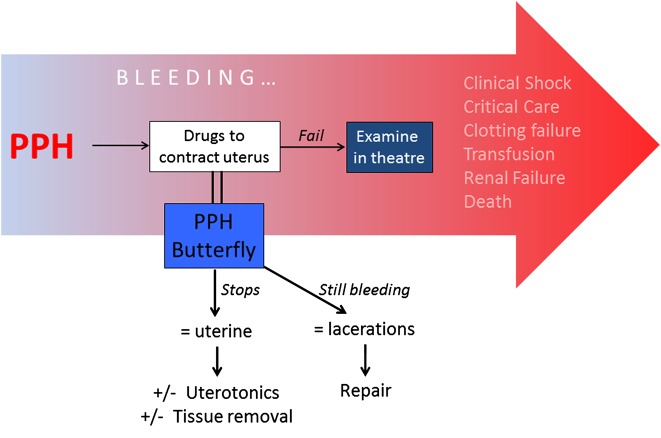
Diagram showing the normal progression of severe PPH. Use of the PPH Butterfly should provide an early diagnosis of the underlying cause of the bleeding while preventing on-going blood loss. PPH, postpartum haemorrhage.

The size and shape of the compression platform was based on that of a shelf pessary, an intravaginal device used for treating uterine prolapse. The depth of insertion was based on the length of a standard Cusco's vaginal speculum (figure 1—shelf pessary and Cusco's speculum combined). This should make uterine compression available for use at a much earlier stage in the PPH treatment process and provide an effective treatment without the need for medicines or advanced diagnostic skills (figure 2).

Once inserted, the uterus is compressed against the PPH Butterfly by a hand externally on the woman's abdomen. The device will be held in place by its handle, which can be wedged against the bed, or be held by the clinician or an assistant.

The tiring nature of traditional BMC is an important issue which limits its effectiveness. In training sessions, we demonstrate this to junior doctors by asking them to compress a book between fist and hand—but they tire rapidly and many are unable to continue this for more than 60 s. However, in keeping with physiological principles of uterine contractions (that generally only occur every 3 min) and blood clots (that take 3–5 min to develop), a pressure period of just 1 min is grossly inadequate except as a temporising measure. To achieve lasting haemostasis, a compression for 8–10 min is needed and this is unlikely to be obtained effectively with standard techniques. To enable prolonged pressure using the Butterfly device, the handle is constructed so as to allow the clinician to stabilise it against the bed once it is inserted. Thus, the clinician's weight can be used to stabilise the device against the bed as well as putting pressure on the uterus—this is a far more ergonomically efficient technique.

**Figure 3:**
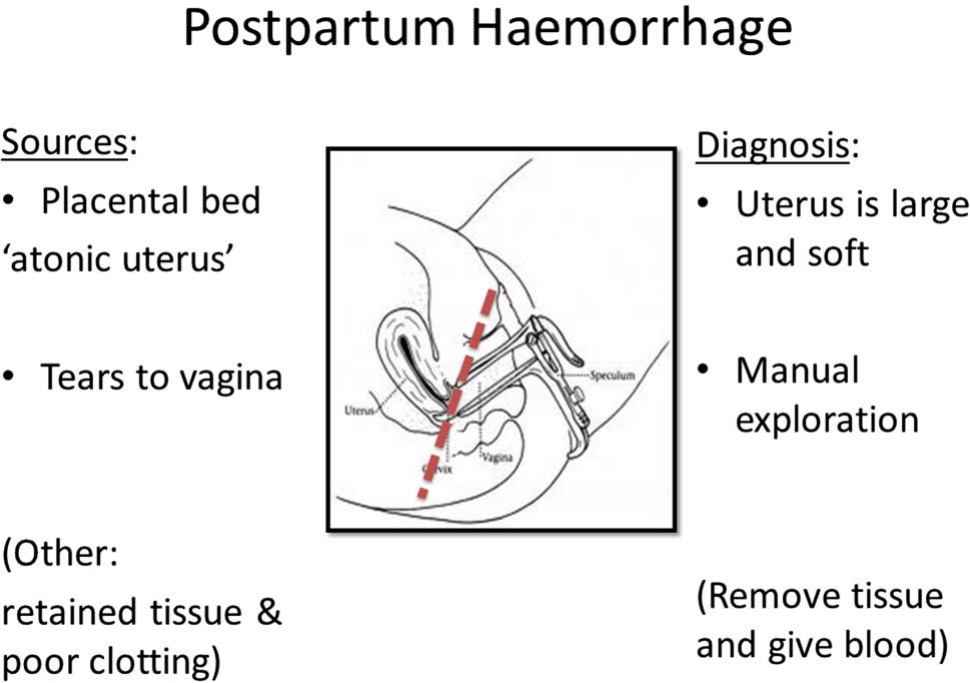
The platform of the PPH Butterfly rests just beneath the uterine cervix (shown as a red dotted line above). Bleeding from above the red line is usually due to uterine atony and will stop abruptly with uterine compression. That from below the red line is from vaginal lacerations and blood loss will continue even with uterine compression. PPH, postpartum haemorrhage.

The PPH Butterfly is also designed to work as a management tool that assists in the diagnosis of primary PPH. There are four well-recognised causes of PPH; uterine atony, genital tract trauma, retained products of conception and bleeding disorders. The most common causes are uterine atony followed by genital tract trauma, but the two are often difficult to differentiate clinically. However, if the bleeding stops with uterine compression, the cause is almost certainly uterine atony: if it continues then it is likely to be from vaginal lacerations (figure 3). The surface of the platform of the device resembles a grill, with multiple holes. This gives a surface against which to compress the uterus, while preventing the trapping of blood and clots above it.

If this concept could be realised, it could transform care in PPH. In high resource settings, it would have the effect of preventing anaemia, as well as the need for anaesthetics, intensive care, blood transfusions and the progression to clotting disorders. If it could also be used in poorer settings where many of these technologies are unavailable, then it could also save many mother's lives every year.

## Methods

The PPH Butterfly was invented by Professor Weeks and John Porter, with the intellectual property held by the University of Liverpool. Following a grant from NIHR i4i funding stream, prototypes were designed and developed with the Department of Medical Physics and Clinical Engineering, University of Liverpool/Royal Liverpool University and Broadgreen Hospitals NHS Trust (RLBUHT). A collaborative relationship was established with Pelican Feminine Healthcare(Cardiff, UK) early in the project. Their input involved an advisory role as part of a twice-yearly Scientific Advisory Group, and optimising the computer-aided design for injection moulding through their design team at Interplex Select Moulds (High Wycombe, UK). The final injection moulding of the model for in vivo testing was undertaken by Proto Labs (Telford, UK) with assistance from Plastribution (Ashby-de-la-Zouch, UK).

As part of the overall project, a series of focus groups were held so as to evaluate the initial concept and design and then review subsequent developments. There were three focus groups: two comprised of mixed groups of midwives and obstetricians, and one comprised of local female members of the public. The first groups explored the groups' initial views of the PPH Butterfly—its aesthetics, purpose, functionality and perceived problems, and sought suggestions for improvements. Further focus groups were held a year later when they were updated as to the progress of the project, saw the outcome of their previous comments and reviewed the progression from the original design.

Updates on the PPH Butterfly as a device, and the project overall, were also presented 6-monthly to the PPH Butterfly Advisory Group. This group comprised of four individuals from the local trust (consultant midwife, consultant obstetrician, head of risk management and a non-executive director), a commercial advisor (from Pelican Healthcare) and the Commercialisation Manager from the University of Liverpool. The group served to provide feedback and advice on a variety of aspects of the project from an outsider's perspective.

## Results

The initial concept is loosely based on combining the shelf pessary and Cusco's vaginal speculum (figure 1). The vagina immediately after delivery is however not in its usual state as it has just been widely distended by the passage of the fetal head. The vagina is therefore relatively distended and loose immediately after childbirth, making it easier to admit a device.

**Figure 4:**
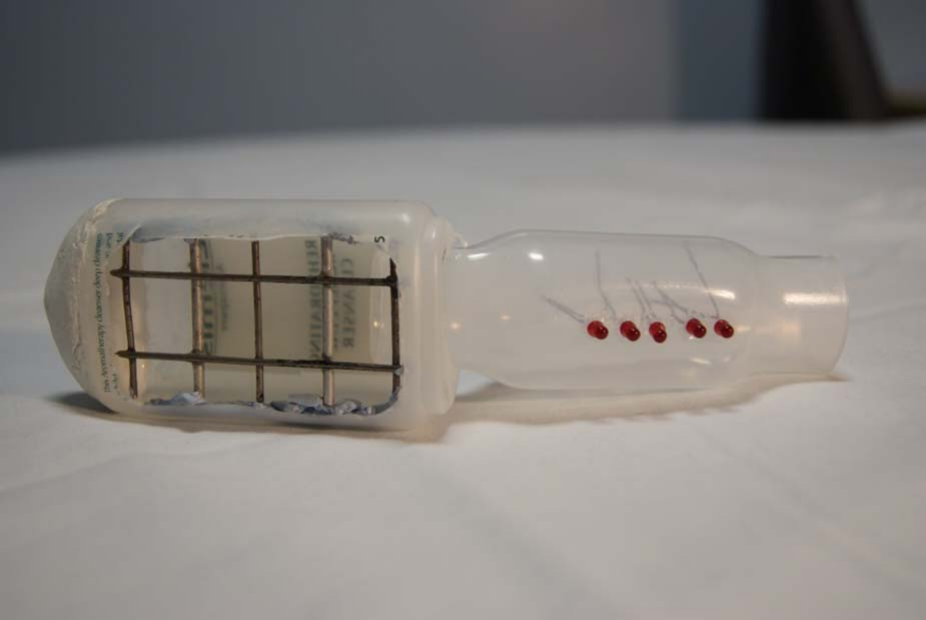
First design using hand cream bottle and asthma spacer.

To date, there have been eight major iterations of the device with subtle adjustments made at each iteration. The first model, made in the kitchen of one of the inventors (figure 4), was made from a plastic hand cream bottle with the side cut out with scissors and a paediatric asthma spacer device. A wire grill was fashioned to place over the cut-out section of the bottle, and the end of the spacer was inserted obliquely into its end. A series of coloured pin heads were inserted into the handle to represent a pressure gauge. The aim was to insert the device into the vagina in an anterioposterior aligned position before rotating it into position in the middle of the birth canal. Its primary function was diagnostic: to separate out blood coming from the uterus and the birth canal. Blood from the uterus would flow through the device and out of the end of the stalk while blood coming from vaginal lacerations would emerge from the birth canal around the sides of the stalk.

**Figure 5:**
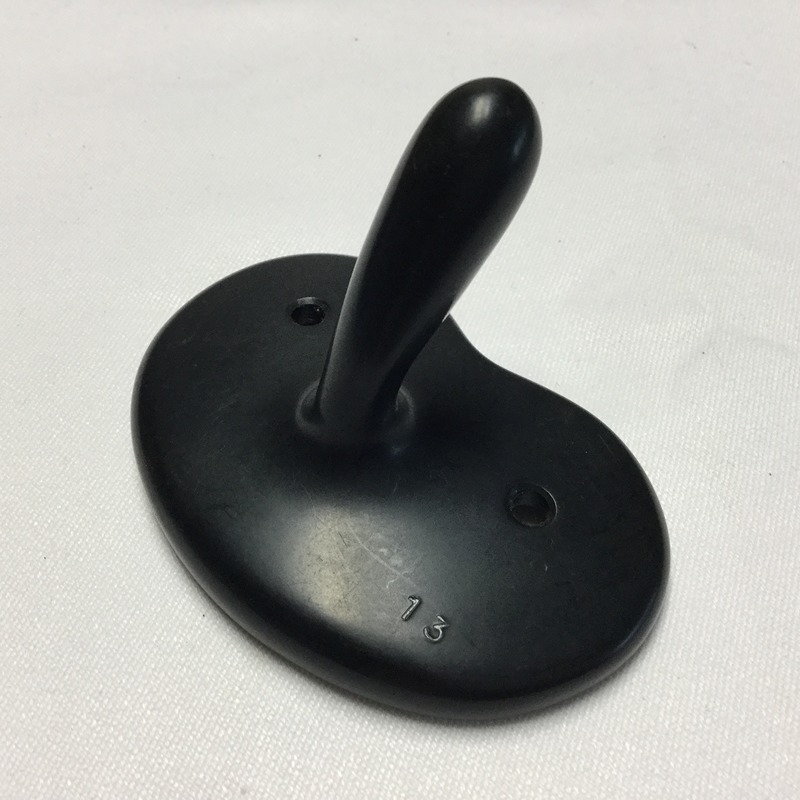
A shelf pessary.

As discussions about the device progressed, it became clear that the BMC function was critical to its success. To be successful, the platform therefore needed to be much larger, similar to that of a ‘shelf pessary’, a gynaecological support device to support a prolapsed uterus in women who are unfit for surgery (figure 5). This would, however, make it much more difficult to insert and then to hold in a stable position during compression. The key design goals were to achieve:

1. A platform that could allow atraumatic compression of the uterus while allowing drainage of blood through it.
2. A system for inserting the platform into the upper vagina without trauma or discomfort and which can be reversed for its removal.
3. A handle to allow the user to hold the platform from outside the birth canal and hold it stable despite up to 100 N pressure on any part of the platform.
4. A safety mechanism to prevent inadvertent overinsertion of the device.
5. A mechanism to assess how much blood is coming from above the device and how much is coming from below it.
6. A mechanism which allows the device to be gripped securely against a surface under the woman's buttocks with minimal effort from the user.

In order to see the effects of each design change, numerous prototypes were made. Initially, the prototypes from hand drawn ideas were made from available materials or adapted from other instruments and machines. However, as the ideas became more developed, the Engineering department moved to computer-aided design (CAD) with 3D printing of APS prototypes. These served to give more of a realistic feel as to how the final device would look. Consequently, these prototype devices were used in a series of manikin studies that were undertaken as part of a student PhD. These studies provided information as to clinicians' opinion of the PPH Butterfly in comparison to traditional BMC. It also assessed the level of pressure that could be achieved over a 5-min period in a randomised trial of the two methods.

**Figure 6:**
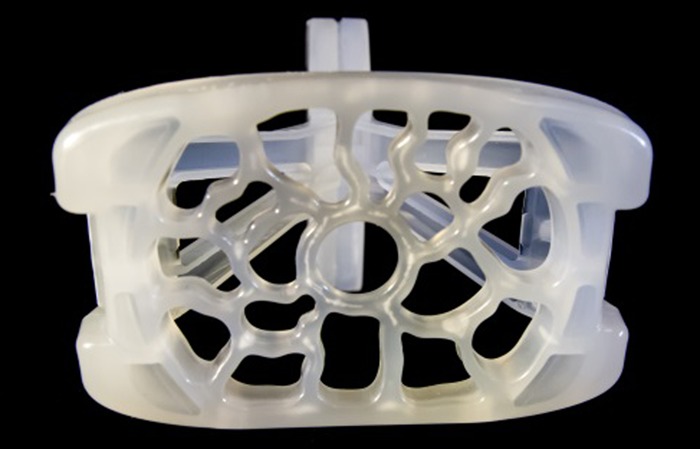
The final design of the platform.

**Figure 7:**
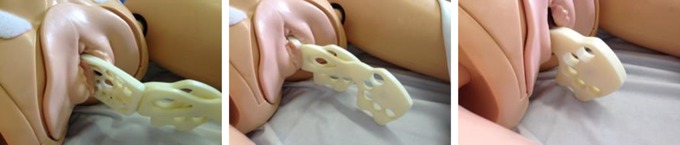
The PPH Butterfly being inserted into the manikin (previous design of the device). PPH, postpartum haemorrhage.

In the final device, the platform shape and size is modelled on that of a large shelf pessary (figures 5 and 6). The addition of a handle enables the user to easily insert the device and align it into its correct position while the large handle limits the depth of insertion. When folded, the device can be slipped longitudinally into the vagina with minimal trauma. Once inserted, the device is unfolded by sliding the two arms of the device over each other so that the uterine platform pivots to face anteriorly towards the maternal abdomen (figure 7). Thus, the surface of the platform ends up perpendicular to the axis of the uterus. On sliding back one handle, the device will return to its original folded state, allowing the device to be removed without discomfort or trauma.

**Figure 8:**
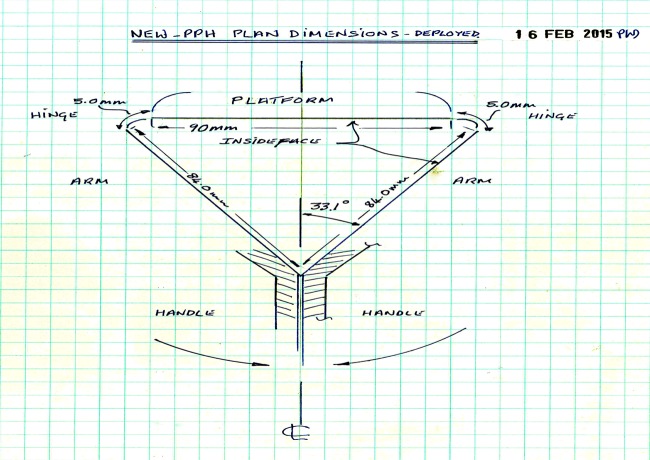
Design diagram showing original dimensions of the PPH Butterfly once folded for use. PPH, postpartum haemorrhage.

The PPH Butterfly is intended as a one-piece injection moulding with living hinges. It is moulded ‘open’ and folded into a stable isosceles triangle for use. When deployed, both handles come into locked apposition and are held locked with the grasp of the fingers. Plan dimensions for the PPH Butterfly triangle form are shown in figure 8. The platform height was originally developed to mimic the size and shape of a fist, although this will be in constant review during human testing.

The device was developed in accordance with the Medical Devices Directive standard BS EN ISO 14155:2011.

There were several areas of the device that required particular attention and revisions.

### Stability of platform (locking)

**Figure 9:**
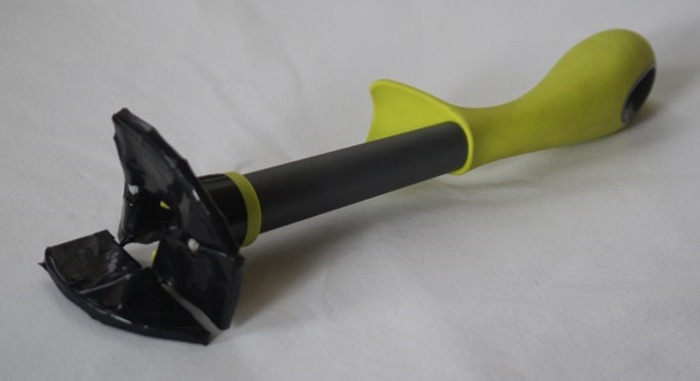
The original design from which the name PPH Butterfly originated. The two wings on either side of the platform fold together for insertion, opening up once within the vagina. Channels on the surface of the wings direct blood down into the drainage channel that runs through the centre of the handle. PPH, postpartum haemorrhage.

Initial designs of the device demonstrated difficulty with the strength and stability of the platform that is the ability of the platform to lock into place once in use. This design feature was key to the functioning and thus potential success of the device. There was also concern that, unless fully stable, the platform could suddenly dislodge/disengage and cause trauma to the vaginal, uterus or cervix. This was a key concern with the initial designs in which the platform was supported by a central handle (eg, figures 9 and 11). The answer to this problem came in the triangular design (figure 8), in which the two supporting arms are continuous with the platform and with the handles, which can be locked together.

**Figure 10:**
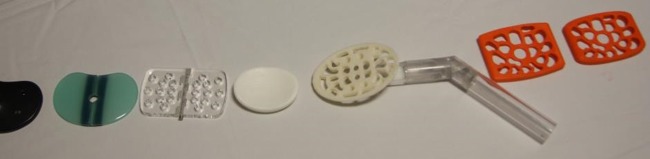
A variety of platform designs (in chronological order from left to right) showing the evolution of the design.

**Figure 11:**
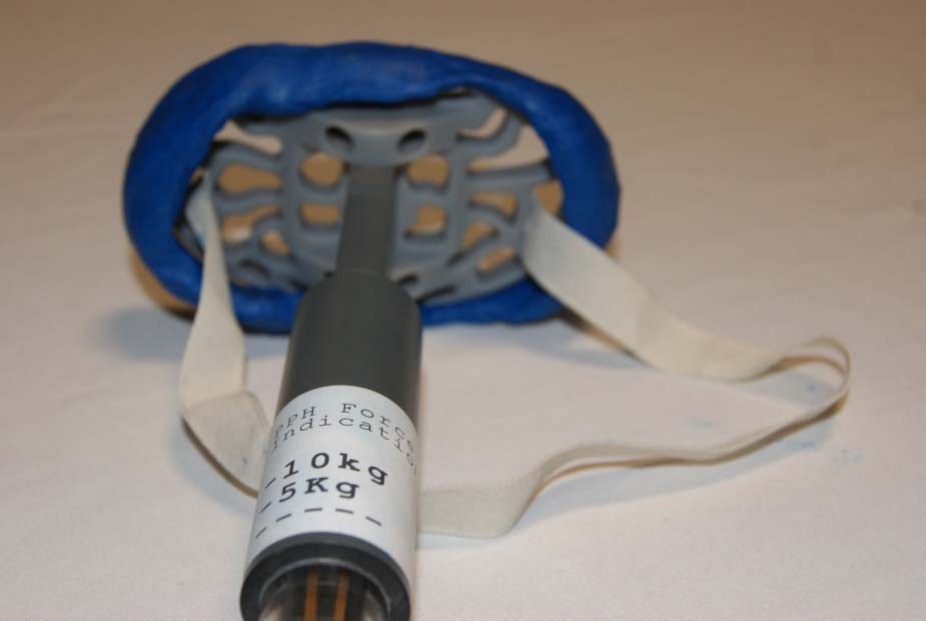
This iteration has a compression gauge as part of the central pillar supporting the platform (note the draft pulley system, designed to pivot the platform from a longitudinal position for insertion into a horizontal position for use).

### Platform design

The platform of the device acts as a surface against which the uterus can be compressed. It was estimated that it needed to be strong enough to support 10 kg of pressure. However, it also needed to have large perforations so as to allow blood and small clots to flow through it. If clots could not pass through the platform, then they could build up above it and give the impression that bleeding had stopped when in fact it was simply being held back inside the uterus.

Initial designs had a central drainage channel that brought blood and clots through the platform and along the central handle (figure 9). There was concern however that a single channel could easily be blocked off by loose cervical tissue and so the decision was made to make numerous large perforations in a platform, large enough to allow small blood clots to pass but leaving enough residual material to support the compression.

Consideration then turned to the platform design, attempting to produce a design that had holes large enough for small blood clots to pass through, but small enough to restrict the passage of the uterus or cervix itself. Any barrier also had to be atraumatic to the lower part of the uterus (ie, without a ‘cheese wire’ cutting effect) and not allow the passage of soft tissue through the perforations which could then swell and be trapped distal to the uterus. Ideally, it would also be aesthetically appealing. Several designs were put to the focus groups, including some where the perforations formed recurring patterns or pictures. An abstract design with variable sized perforations with a depth of at least 4 mm was finally settled on which remains in use in the first in vivo device (figure 6).

The size of the platform was not easy to determine but was based on the dimensions of a flat hand and clenched fist as used in BMC. The birth canal immediately after childbirth has just been widely distended with the passage of a baby's head. This is typically 10 cm diameter at the time of birth, and the resulting distension of the birth canal is thought to remain for several hours after birth. It is this distension that gives space for the insertion of a clinician's flat hand into the vagina and space to clench it into a fist for BMC. These dimensions were therefore chosen for the initial in vivo version of the device.

Subsequent testing of the device in five normal postnatal women revealed that the platform design was too large. For subsequent human tests, two sizes were therefore produced with reduced dimensions of 8.0×5.9 cm and 6.9×5.0 cm.

### Living hinges

To facilitate folding of the arms, the device has ‘living hinges’ at either side of the platform. This is a section of the device between the platform and the arms where the plastic suddenly narrows to 2 mm thickness, thus producing a natural point at which the device will crease. This allows the device to be moulded in a single part, reducing its complexity and price. Although a living hinge is strong, there is the potential for tearing after repeated folding. Initially, however, these hinges are strong and carry no risk of tearing during the single use that the device is made for.

**Figure 12:**
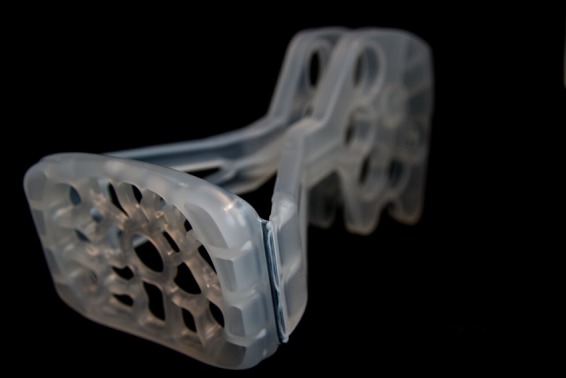
The initial hinge design—strong but with a sharp leading edge that could result in trauma on insertion.

**Figure 13:**
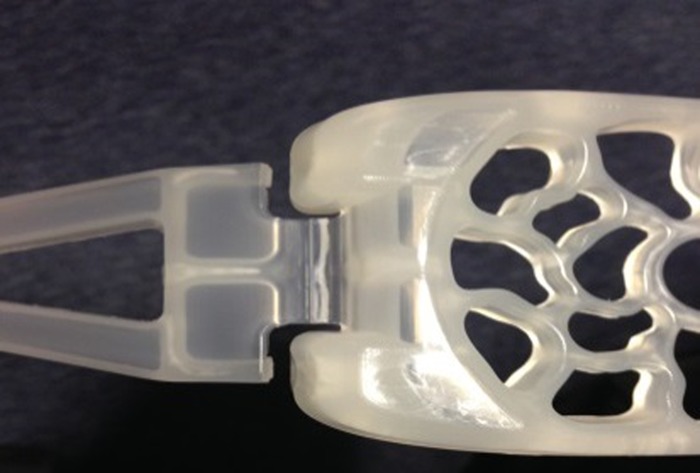
The redesigned shorter hinge. The sharp edges of the folded living hinge are now recessed behind extensions to the platform. Once folded for use, each arm has two pedicles lateral to the hinge that rest over the outer edges of the platform. As soon as the hinge starts to ‘give’ with pressure, these pedicles push tight against the platform thus ensuring strength and stability.

The first injection-moulded prototype had a living hinge that extended right along both sides of the platform. Once folded, this formed a sharp ridge along the leading edge of the platform, which would be likely to cause trauma to the vaginal walls during insertion (figure 12). The platform edge was therefore redesigned to recess the living hinge behind two lateral pedicles (figure 13). This gave a smooth leading edge, but also gave the advantage of added stability once in the compression position. In the first injection mould, the total compression force was borne by the living hinges. In the redesign, however, the pedicles took the full force of the compression as soon as there was any give in the hinges. This gave greater stability and weight bearing ability.

### Minimising trauma

In order to aid the ease of insertion and minimise any potential trauma to women, the device edges have been rounded as much as possible. A plastic was also selected for manufacture that had a smooth surface and could be lubricated with one of the water-soluble gels commonly used in hospitals. In the injection-moulded prototypes, flashes and joint ridges were smoothed out by hand prior to sterilisation and clinical use. The change in hinge design (see above) also minimised trauma.

### Preventing overinsertion

During formal risk assessment, it was identified that there was a danger of inexperienced users inserting the device too far into the birth canal and so damaging the top of the vagina. It was therefore planned that a guard or shield would be added halfway up the handle of the PPH Butterfly to act as a safety mechanism by indicating to clinicians how far the device could be inserted safely. Its distance from the platform was based on the length of an adult Cusco's speculum, an instrument for inspection of the cervix which is in common hospital use. It was subsequently decided that a guard could cause confusion and that it would be better to simply increase the size of the handle of the device. This not only clearly indicates the limit of insertion, but it also prevents overinsertion through its large size (figures 7 and 9).

### Handle—inclusion of finger holes

**Figure 14:**
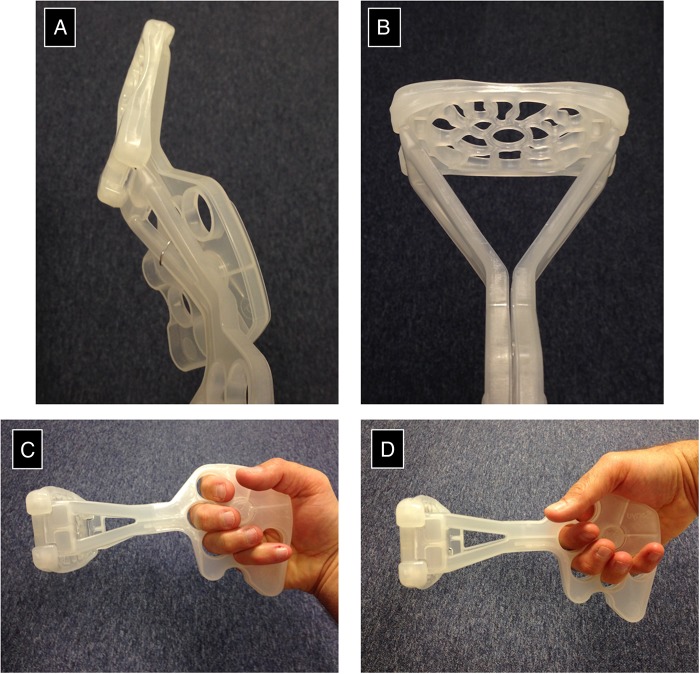
(A–D) The final iteration of the device. The device is folded flat for insertion (A) and then the handles brought together so as to create a stable triangular structure to support the weigh-bearing platform (B). The handles are held together by complimentary protrusion and recesses in the handle as well as by the finger grips. The device can be held in a longitudinal fashion (C) to mimick standard bimanual compression. For women lying on a bed, the handle can be stabilised from above (D) so as to wedge the handle onto the bed. This mechanism should be less tiring for the clinician as he/she can use their body weight to put pressure on the uterus as well as to hold the device in position.

The Butterfly handle varied in material, shape and length over the course of the design process. Concerns were raised as to the difficulty and discomfort that would be experienced when having to grip the handles for the length of time that would be necessary to compress the uterus effectively. The solution came with the inclusion of five finger holes in a wide handle for the user's fingers to go through (figure 14). Placing the user's fingers through the holes not only held the two sides firmly together even in the presence of blood or other body fluids, but also gave a stable grasp for the user. The inclusion of the finger holes allows for variation in the way that the device can be held by the user: the device can either be held ‘in-line’ with the Butterfly stem in continuity with the forearm when in use or the hand can hold the handle from above. This allows the user to push the handle into the bed beneath the woman to hold it stable. This should provide a less tiring position for the user as his/her body weight can be used to simultaneously compress the uterus and hold the device in place.

### Material choice

A material had to be chosen that is suitable for internal use in women, while being robust, smooth and economical. Polypropylene was an obvious choice for this. The TOTAL product PPM H250 was chosen as it is ‘a narrow molecular weight distribution homopolymer with a Melt Flow Index of 25 g/10 min, recommended for the manufacture of medical packaging and medical devices, to the exclusion of implants’ (TOTAL, 2013).

### Naming the device

The ‘PPH Butterfly’ name was initially chosen because the earlier incarnations of the device had operated in such a way that when opened, the platform of the device resembled a pair of wings, like that of a butterfly. When the design changed to a platform with folding arms, it was felt this resemblance was lost, and a change in name to ‘PPH Shelf’ (after the shelf pessary on which the device is based) was considered. However, this option was not at all popular with the focus groups, and it was decided to retain the original name of ‘PPH Butterfly’.

### Blood collection

The original concept of the device was that it would divide the genital tract into two and separate any blood coming from above the device from that coming from below. This can be seen in the very first home-made prototype (figure 4) where blood from above the device passed through the grill and down the handle. This was later modified so that the platform had channels along which blood flowed into a central stalk (figure 9). The realisation that a central hinge in the platform would be a point of weakness led to the production of a solid platform (figure 10). This would need to be twisted to allow its longitudinal insertion into the birth canal, complicating the drainage of blood. A suggestion came from a focus group to attach a blood collection bag to the rear of the platform. The aim of the bag was to collect any blood coming through the platform and give a physical recording of the amount of blood that had been lost while the device was in use. It would also have enabled a visual assessment and quantification of the volume and source of the blood loss. While an attractive option, the bag would have added complexity to the manufacture, and there were fears that it would be compressed in the birth canal and prevent blood entering it. Furthermore, an assessment of the source of the bleeding could be made without the bag: if the bleeding stopped as the uterus was compressed, then you would know the bleeding was uterine in origin. If it continued during uterine compression, then you would know that it was from vaginal lacerations. Consequently, it was agreed that the bag provided considerable complexity for little added benefit and it was removed.

### Compression gauge

Earlier in the design process consideration was given to the inclusion of a compression gauge that would provide feedback as to the level of pressure that was being used to compress the uterus. This can be seen in figure 11.

This was to be sited along the handle and would have provided biofeedback to the user as to whether the pressure was adequate. However, when measurements were taken of the pressure exerted by 10 experts on the uterus of a manikin during BMC, the pressures were found to be highly variable. It was not therefore possible to provide a standard pressure, and the compression gauge was abandoned.

### Device assessment and risk management

During initial BMC measurements in a manikin with prototype PPH Butterfly devices, peak levels of 42 N were recorded.

Subsequent stress testing of real prototypes (PPH Model (2) A, B and C), as part of the Medicines and Healthcare Products Regulatory Agency (MHRA) submission, demonstrated devices could withstand forces of up to 902, 1140 and 1122 N, respectively, without buckling.

Feedback was obtained throughout the design period from clinicians who compared the PPH Butterfly device and BMC in an obstetric manikin. In initial studies, a commercial potato masher was used as a substitute for the device but this was replaced by 3D printed prototypes once available. A final cross-over study compared the uterine pressures generated by the device and bimanually in 20 doctors who had previous experience of undertaking BMC and 22 midwives who had no experience of it. The study measured maximum pressure generated as well as total pressure over a period of 5 min. There was no difference in the mean or maximum uterine pressures generated using BMC or the PPH Butterfly and no difference between the two groups.

## Discussion

Now that the design of the PPH Butterfly is complete, the next step in the project is to test the device in postnatal humans—normal and in those with bleeding. Initial tests on normal postnatal bleeding will assess the size of the platform and handles as well as the mechanism for insertion. Once this has been explored, it will then be necessary to determine that the PPH Butterfly can effectively manage PPH.

If successful, the PPH Butterfly has the potential to make a significant impact on PPH management. This would be particularly true within the developing world, where there is limited access to resources. The PPH Butterfly has been designed specifically with these countries in mind, with particular attention being paid to the overall cost of the device, which has been kept as low as possible, while not compromising the design. While it is designed to be single use and disposable in the UK, there are also plans to make a version that can be sterilised and re-used for low resource settings. This will allow it to reach a much wider number of women than would be possible if it only withstood one use.

The use of BMC to treat PPH is limited by its invasive nature and how tiring it is for the clinician. These factors currently limit its use to those who are bleeding very heavily where its efficacy is likely to be the lowest. Consequently, its effectiveness is not demonstrated, which in turn leads to a further reluctance from clinical staff to use it as a method of PPH management.

Suggestions have been made by the design team and members of the focus groups for ways in which the device could be enhanced, such as the inclusion of a light on the handle, and the addition of a soft covering or local haemostatics to the platform. While such additions may enhance the device, they are not necessary in relation to the basic function of the PPH Butterfly and instead could be seen as potential future ‘add-ons’.

Discussions with focus groups during the course of the project have provided the project team with real time feedback as to the design of the device and possible ideas for its development. Currently, the device needs to achieve basic functionality, while being safe and cost-effective. Once these aspects have been proven, it may be possible in the future for there to be some ‘add-ons’ to the device, at an additional cost. The focus groups facilitated the opportunity to view the device from a different perspective. Having focus groups comprised of clinicians and members of the public allowed the project team to see past their own enthusiastic viewpoint and also gave an appreciation of the way in which the PPH Butterfly may be seen by those women it could be used on. This was useful prior to beginning any recruitment to the human studies of the project, as it allowed the project team to carefully consider the best way in which to identify and approach women, as well as how to project the PPH Butterfly and the study itself, in order to increase the likelihood of participation.

## Conclusions

The PPH Butterfly is a novel device designed to treat PPH through uterine compression. It is built to be less invasive and less tiring than traditional BMC. Clinical assessments are currently being undertaken to determine its safety and effectiveness in PPH management.

## References

[1] Say L, Chou D, Gemmill A, et al Global causes of maternal death: a WHO systematic analysis. Lancet Glob Health 2014;2:e323–33. 10.1016/S2214-109X(14)70227-X 25103301

[2] Alkema L, Chou D, Hogan D, et al Global, regional, and national levels and trends in maternal mortality between 1990 and 2015, with scenario-based projections to 2030: a systematic analysis by the UN Maternal Mortality Estimation Inter-Agency Group. Lancet 2016;387:462–74. 10.1016/S0140-6736(15)00838-7 26584737 PMC5515236

[3] Lori A Hidden suffering: disabilities from pregnancy and childbirth in less developed countries. Washington (DC): Population Reference Bureau, 2002 http://www.prb.org/pdf/HiddenSufferingEng.pdf

[4] WHO. Making pregnancy safer. Reducing the global burden: postpartum haemorrhage. Geneva, Switzerland: WHO, 2007.

[5] WHO. WHO recommendations for the prevention and treatment of postpartum haemorrhage. Geneva, Switzerland: WHO, 2007. 23586122

[6] WHO. WHO Guidelines for the management of postpartum haemorrhage and retained placenta. Geneva, Switzerland: WHO, 2009. 23844453

[7] Green L, Knight M, Seeney FM, et al The epidemiology and outcomes of women with postpartum haemorrhage requiring massive transfusion with eight or more units of red cells: a national cross-sectional study. BJOG 2016;123:2164–70. 10.1111/1471-0528.13831 26694742

[8] Breathnach F, Geary M Uterine atony: definition, prevention, nonsurgical management, and uterine tamponade. Semin Perinatol 2009; 33:82–7. 10.1053/j.semperi.2008.12.001 19324236

[9] Sheehan SR, Montgomery AA, Carey M, et al Oxytocin bolus versus oxytocin bolus and infusion for control of blood loss at elective caesarean section: double blind, placebo controlled, randomised trial. BMJ 2011;343:d4661 10.1136/bmj.d4661 21807773 PMC3148015

[10] Widmer M, Blum J, Hofmeyr GJ, et al Misoprostol as an adjunct to standard uterotonics for treatment of post-partum haemorrhage: a multicentre, double-blind randomised trial. Lancet 2010;375:1808–13. 10.1016/S0140-6736(10)60348-0 20494730

[11] Balki M, Erik-Soussi M, Kingdom J, et al Oxytocin pretreatment attenuates oxytocin-induced contractions in human myometrium in vitro. Anesthesiology 2013;119:552–61. 10.1097/ALN.0b013e318297d347 23676375

[12] Weeks AD, Neilson JP Rethinking our approach to postpartum haemorrhage and uterotonics*.* BMJ 2015;351:h3251 10.1136/bmj.h3251 26156874

